# Supplementary material for: The combined effect of eye movements improve head centred local motion information during walking
Source: PLoS One. 2020 Jan 30;15(1):e0228345. doi: 10.1371/journal.pone.0228345 (PMC6992003; doi:10.1371/journal.pone.0228345)
Supplement: Attachment — Submitted filename: DurantandZankerPLOS Response to Reviewers.docx [file pone.0228345.s001.docx]

We would like to thank the reviewers for their helpful and often detailed comments, please see below, how we have addressed these. The line numbers included in our response below correspond to the version of the manuscript with no tracked changes.

Reviewer #1:

This manuscript describes a study that primarily sets out to determine whether extraction of motion cues from a moving scene can be improved by taking into account eye-movements. Because eye-movements serve a number of functions it is somewhat hard to determine whether the focus of the study is to examine stabilising eye-movements, scanning eye-movements or a combination of the two. Whilst I like the general idea (to look at eye-movements during real-world walking to determine how they might be used to help extract motion information) my sense after reading the paper is that the results mainly confirm what is already known: that some eye-movements have a useful role stabilising the retinal image when walking, but other eye-movements make it harder to extract motion signals from the retinal image compared to pure target fixation.

Response

The focus of the study is to examine the combination of the two types of eye movements, as we have now additionally clarified on line 107. The comparison between the fixed and natural conditions does indeed show that other eye movements than stabilising ones make it harder to extract motion signals (added this to clarify on line 377), but this is not our main comparison and this is the less interesting result. Our main comparison (re-stated as such and rearranged in the writing to make clearer on line 358) between the head centred frames (where eye movements do not affect the input) vs the retinocentric frames (which have the combined effect of these two type of eye movements) we find an overall improvement in retinocentric coordinates, showing that although some of the eye movements may not be helpful, overall optic flow information is improved by them.

Reviewer #1:

The approach in the paper is to ask participants to walk to a target whilst wearing an eye-tacker, then extract motion signals from the head-mounted scene camera video, and compare this to when motion-signal extraction uses an eye-movement transformation. Contained within the eye-movement transformations are a combination of stabilising and scanning components. The authors manipulate these components by having two different fixation conditions: ‘Fixate’ (where participants walked whilst smoothly tracking/fixating a target, so this predominantly contains stabilising eye-movements) and ‘Natural’ (where participants walked and looked to the target, but were encouraged to look around freely, so this presumably had more scanning eye-movements whilst also containing stabilising eye-movements). The authors demonstrate that they were best able to extract direction of motion information from scene camera images using stabilising eye-movements, but that ‘Natural’ eye-movements were better than no eye-movement signal. Because the characteristics of ‘Natural’ fixations seem to be unquantified it is hard to see how the authors can achieve their main goals: examining the “trade-off between stabilising and scanning the scene” (line 79-80), determining ‘how much do eye-movements distort the optic flow pattern on the retina?’ (line 102) and aiming ‘to investigate if the net result of eye movements is to reduce … noise’ (line 126). Each of these goals would seem to require a more detailed examination of what eye-movements were being generated in 'free' conditions (and quantifying them) during their walking tasks in order to then determine their contribution to the retinal patterns produced.

Response

We would again say, that we aim to look at is the combined effect of these eye movements rather than aiming to separate them out. We agree though that it is important to check that in the natural condition participants did not simply keep their eyes fixed on the target. Although in the section under Eye movement statistics we did show the variation in location between target and gaze position as well as the correlation between target position and a gaze position as a way to quantify how much the eyes scanned around, we admit that this is an indirect measure but does show that participants were freely moving their eyes in the ‘natural’ condition as there is large variation between the eye position and the target position. We have now added a measure of ‘time on target’ vs ‘time off target’ to better quantify these as requested, with an additional figure (Figure 2) illustrating how these time periods were defined.

Other major queries:

Reviewer #1
Q1. There needs to be a fuller up-front rationale/explanation for the visual field sampling. The manuscript does mention this point in the discussion (line 371) but merely states that this is a limitation without giving the reader a sense of how this might impact upon generalisation of the findings. When reading the manuscript I had the following questions: There is a difference between the field of view of the camera and the actual visual field of participant - the 82deg horizontal camera view is much less than the visual field of the observer, leading to reduced peripheral signals. Because optic flow motion vectors are usually larger in the periphery could this not have led to underestimation of the impact of eye-movements on detecting self-motion? Linking this to line 198/199 the MS mentions ensuring there are ‘equal areas of the visual scene’ and then line 201-202 ‘cropped in horizontal dimension’ to ‘540x540 pixels’ – what field of view does this effectively reduce the visual field down to? My calculations based on line 213 (8 pixels = 0.3deg) would suggest this is 20.25 x 20.25deg? (in the discussion it mentions around 23 deg) I think it would be good to mark this effective size on Figure 1 to give the reader a sense of the areas of the scene actually contributing to the current analysis since currently there is a slightly misleading sense that the whole scene image is contributing (whereas much of the peripheral information won’t be I think).

Response

We apologise, we have found a mistake in our calculations here. In fact we reduced the resolution of the input frames by half, keeping the vertical FoV and making them square so where originally 1920 x 1080 was 82 deg across (and 46 deg vertically), in the input images for the model 540 pixels was 46 deg. We have corrected 8 deg to 0.6 pixels and in the Discussion to 46 deg. The confusion is due to some distances in pixels being reported being calculated in the raw output frames (1980x1080) or in the input frames for the motion calculation model (half resolution 540x540). We have clarified in the manuscript if we are referring to pixels in the raw output frames or in the input frames for the model (line 200, 218, 246, 274, 310, 320).

To answer the point on limited FoV we have added in the Discussion (line 447):

A large amount of research suggests that more peripheral motion cues are important for detecting optic flow, suggested by the very large receptive fields found in MST, sensitive to redial flow patterns [32] and corresponding psychophysical evidence for integration of radial flow over visual areas of up to 70 degrees [33]. However, some research also suggests that central vision is very important for using optic flow for heading estimation [34]. In our case this limited part of the scene contained enough information for successful extraction of the FOE, in that the motion information yielded smaller template matching errors than random motion would. However, our method does not allow us to estimate the effect of eye movements on more peripheral motion signals, which may be different

Reviewer #1
Q2. Lines 202-204. Does stating the average luminance values (~0.5) on a scale of 0-1 of possible image values have any useful meaning? Presumably the outdoors scene was actually much brighter than indoors, but the camera recorded images of similar luminance across scenes due to reduced aperture size (or other scaling methods to prevent overexposure and thereby keep luminance values broadly similar)? Is that the point of the statement – to show that the recorded scene images were similar? If so providing average luminance values for both scenes might be necessary? I suggest giving the reader an idea of why this information is presented to aid interpretation.

Response

Have added on line 235 “There was no difference in the mean luminance level of two types of image sequences, it was the same at 0.48, and the s.d. was the same at 0.01 (on a scale of 0-1 possible image values) for both.”

Reviewer #1

Q3. I think there needs to be greater consideration of the particular types of eye-movements (i.e. saccades) that would be affected by the analysis/processing methods. Line 220 suggests that high speed changes are filtered out. This issue is somewhat acknowledged in the discussion but I am still unclear whether pooling across 10 frames (~400ms) removed all saccades but left other eye-movements intact, or whether other rapid eye-movements (e.g. OKN) might also be removed in this pooling. How might this affect conclusions about the role of eye-movements given that saccades will predominantly be supporting scanning behaviours, whereas stability would be supported by slower/lower frequency tracking eye-movements.

Response

We agree, this is in fact why we think that eye movements overall improve the signals. We have added a sentence in the Discussion (line 427):

The temporal scale used in our model may have reduced the effects of fast saccade type eye movements more than slower pursuit-like stabilising eye movements, suggesting that at the temporal scale needed to extract informative optic flow information, large scanning eye movements contribute less to the extracted motion signal.

Reviewer #1

Q4. Template matching errors in the region of 25-70 degrees are reported but I initially struggled with interpreting these values. Lines 304-307 are very helpful in clearly explaining what the values mean. I would recommend adding to the Figure 4 caption an explanation that matching errors of 90 degrees would indicate a random motion pattern (and therefore no match).

Response

Have now added this to the caption of Figure 5 (which used to be Figure 4).

Reviewer #1

Q5. Methods (Line 146-166). The authors need to clarify what sorts of trajectories were taken when walking. Given the images in Figure 1 and the description of the corridor I presumed that straight line walking was produced in both environments? (there are hints in various places this might be the case). However the outdoor scene seems to have the target placed off to the side of the path (figure 1). Did participants walk straight along the path or straight towards the target? Was the target intentionally offset with respect to the trajectory to add additional gaze rotations? This aspect of the design was quite unclear to me until I found the videos. These are much richer than the current manuscript descriptions but should not be relied upon to explain how the task was designed, especially since the rationale behind the design choices could be clearer. I imagine the authors did not record spatial position during walking (other than via the scene camera) but unless otherwise constrained people tend to walk in a straight line to complete the described tasks. This issue could be important since some of the discussion in the literature around use of optic flow has centred around whether retrieving heading from flow is necessary when steering curved paths and fixating points on your future path (rather than walking straight and looking straight ahead). Indeed the MS refers to this issue in passing in the discussion. I think it is also important since the main conclusions are that eye-movements improve the quality of the motion signal for the extraction of the direction of heading (compared to head centric coordinates). It is unclear to me that this would still be true when tracking points on the ground when travelling at speed around a bend in a road.

Response

We have now clarified (lines 130, 152, 156, 159, 496, 500) that they head in a direct, straight trajectory, so this is the situation we are describing.

Reviewer #1
Minor:

Title: The title states that eye-movements improve extraction of local motion information during walking, but this seems misleading (since it's not clear what the improvement is relative to) - the reader’s natural inference may incorrectly be that the authors are comparing free gaze conditions (lots of eye-movements) versus gaze fixation (reduced numbers/types of eye-movements) in this title.

Changed title to: The combined effect of eye movements improve head centred local motion information during walking

Line 165 – it's not clear what ‘3 measurements’ could entail (3 recorded frames, 3 full trajectories without any data loss? Greater precision here would be helpful.

Corrected (line 170): of at least 3 trajectories (out of the 6 trajectories recorded over both directions for each condition).

Line 233 – ‘patter’ should be ‘pattern’

Corrected

Line 296 – ‘in sync’ should probably no be abbreviated (i.e. ‘in synchrony’)

Corrected

Line 315-318 – I found these statements about the results a bit hard to follow because of inconsistent labelling, e.g. ‘kept their eyes fixed in both frames of reference’ – for clarity please use consistent labels throughout. Lines 309-371 refer to: “scenes, task conditions, coordinates” but elsewhere other labels (“fixed, frames of reference”) are used.

Corrected, line 357-375

Line 320 - ‘form’ should be ‘from’

Corrected

Line 330 – ‘the reduction in noise’ – which reduction? Not clear from the context.

Line 372 changed: this reduction in noise from head centred to retinocentric coordinates is meaningful.

Line 356 – ‘broadband luminance-defined stimuli’ Could the authors clarify what they mean by this?
Clarified (line 422): stimuli containing broadband luminance-defined motion

Line 376 – “Successful extraction of FoE”. I’m not sure the authors provide a way of determining success in extraction of the FoE. Is any value of template matching error less than 90 degrees success? Or does template matching need to be close to zero? They demonstrate improved accuracy of extraction in certain conditions but in the literature a criterion value (e.g. gauging heading to within 2 deg or less) has tended to be needed to determine whether heading is gauged with sufficient accuracy to guide an action.

Detail added (line 453): in that the motion information yielded smaller template matching errors than random motion would.

Line 415 – “driving fast eye-movements”. Could you be more specific? Do you mean driving (a car) or do you mean something like optokinetic nystagmus?

Simplified: Line 493 Whilst this article concerns itself with the use of optic flow for guided walking it is worth noting that there are other uses of optic flow such as postural control and flow-parsing.

Line 418 – “regular” – I think you mean smooth or flat?

Changed to: In our study the walking terrain was smooth and flat

Reviewer #2: This paper is about an important question - whether eye movements improve local motion information during walking. The optical situation is this. Consider a rectangular frame of reference, centered on O the nodal point of an eye and attached rigidly to the head of an observer. The frame of reference will move relative to the environment as the observer walks through the environment. There are six components of motion of the head frame of reference relative to fixed axes in the environment. These are three rotary components, and three translatory components. At any instant, if the rotary components are zero, the optic velocity field at O will be expanding radially, and the centre of expansion will correspond to the direction relative to the environment along which O is moving at that instance. In this case, the optic velocity field would afford clear information about the direction of heading of O. However, if, as will usually be the case, the rotary components of the head frame of reference are not all zero, then the optic velocity field at O will be the vector sum of the radial optic velocity field due to the translatory movement of the head frame of reference, and a rotary optic velocity field due to the rotation of the head frame of reference. In this case, the optic velocity field would afford noisy information about the direction of heading of O. The visual system could in principle deal with this problem by counter-rotating the eye in the head to cancel out the rotary movement of the head frame of reference. To what extent the eye can do this is an empirical question which I had hoped the paper would be answering. Maybe the 2DMD analytic method they used is directed in that direction, but it is unclear that it is, from the scant outline of the analytic method given in the paper, and the scant results presented, which do not properly address the problem. This is a great pity, because I for one would very much like to know how the active visual system works in such everyday activities as walking around the environment.

Response

We agree that the extent the eye can counter rotate to correct for head movements is an interesting empirical question. This could be answered precisely in controlled lab conditions. The question of this paper is slightly different and we are sorry that the reviewer was disappointed. We hope the new title makes it clearer what the article will contain. The question was about the combined effect of all types of eye movements including these counter rotations during naturalistic real life walking.

We have added a paragraph (line 389) in the discussion to address the reviewer’s concerns that our focus is too narrow and added some of the description of the optical situation which was helpfully provided by the reviewer also in the intro at line (and also 41). We are happy to add more detail in the methods and results with some suggestions of where these are needed.

“Whilst walking the rotary components of the head frame of reference are not all zero, and thus the optic velocity field will be the vector sum of the radial optic velocity field due to the translatory movement of the head frame of reference, and a rotary optic velocity field due to the rotation of the head frame of reference. Thus the optic velocity field affords noisy information about the direction of heading. The visual system could in principle deal with this problem by counter-rotating the eye in the head to cancel out the rotary movement of the head frame of reference. However, in addition to these counter rotations the eyes are also scanning the visual field for relevant information, which may hinder the ability to extract information about the direction of heading from the velocity field. Whilst mobile eye tracking offers the opportunity to study the interaction of body, head and eye movements in navigation there are considerable restrictions of the visual field as we elaborate below and only a rather slow and unreliable rotation signal of head movement is provided by the eye tracker gyroscope and accelerometer.

Given these theoretical and practical challenges, our approach is to restrict ourselves to a very confined question – the effect of gaze position during the approach of a target on the overall quality of optic flow information picked up by the retina from the environment, as compared with the scene camera information, where gaze position is not taken into account. This is a partial step to understanding the comprehensive processing mechanisms of optic flow in human agents moving through a natural environment.”
